# The unicompartmental knee is the preferred side in individuals with both a unicompartmental and total knee arthroplasty

**DOI:** 10.1007/s00167-019-05814-7

**Published:** 2019-11-28

**Authors:** Anatole Vilhelm Wiik, Dinesh Nathwani, Ahsan Akhtar, Bilal Al-Obaidi, Robin Strachan, Justin Peter Cobb

**Affiliations:** Imperial College London, Charing Cross Hospital, Fulham Palace Road, London, UK

**Keywords:** Knee, Arthroplasty, Unicompartmental, Gait, Treadmill, Biomechanics

## Abstract

**Purpose:**

To determine the preferred knee in patients with both one total and one unicompartmental knee arthroplasty.

**Method:**

Patients simply with a unicompartmental (UKA) and total knee arthroplasty (TKA) on contralateral sides were retrospectively screened from three senior knee surgeon’s logs over a 15 year period. Patients safe and free from other diseases to affect gait were approached. A total of 16 patients (mean age 70 ± 8) agreed to ground reaction force testing on an instrumented treadmill at a fair pace and incline. A gender-ratio identical group of 16 healthy control subjects (mean age 67 ± 10) and 16 patients with ipsilateral medial knee OA (mean age 66 ± 7) were analysed to compare.

**Results:**

Radiographically the mode preoperative Kellgren–Lawrence knee grade for each side was 3. Postoperatively, the TKA side had a mean coronal femoral component alignment of 7° and a mean tibial coronal alignment of 89° with a mean posterior slope of 5° in the sagittal plane. The UKA side had a mean coronal femoral component alignment of 7° and a mean tibial coronal alignment of 86° with a mean posterior slope of 4° in the sagittal plane. In 7 patients, the TKA was the first procedure, while 6 for the UKA and 3 done simultaneously. Gait analysis demonstrated in both walking conditions the UKA limb was the preferred side through all phases of loading (*p* < 0.05) and nearer to normal than the TKA limb when compared to healthy controls and patients with knee OA. The greatest difference was observed between the transition of weight acceptance and midstance (*p* = 0.008), when 22% more load was taken by the UKA side.

**Conclusion:**

By using a dynamic metric of an everyday activity, a distinct gait difference between differing arthroplasty types were established. A more natural loading pattern can be achieved with unicompartmentals as compared to total knees.

**Level of evidence:**

Retrospective comparative study, Level III.

## Introduction

In the United Kingdom as much as 47% of patients presenting with symptomatic knee osteoarthritis (OA) have single compartment disease [5, 23, 29]. The great majority are offered and accept total knee arthroplasty (TKA) [17] which will result in the removal of a potentially functional anterior cruciate ligament (ACL) together with otherwise competent compartments. Unicompartmental knee arthroplasty (UKA) was introduced as an alternative to preserve these anatomically coupled parts with a unique working relationship for knee motion [16]. While medically safer in terms of myocardial infarction, stroke, deep vein thrombosis, deep infection and early death [4, 6, 8, 13], the rate of UKA revision surgery is still three times higher than TKA in the long term [6, 13, 17]. An ongoing national pragmatic randomised total or partial knee arthroplasty trial (TOPKAT) has only found a small statistical functional difference in favour of UKA which questions the clinical relevance and continues the quest to determine if a skeleton sparing procedure with higher revision is worthwhile [2]. The clinical question which continues to be unanswered is the patient-centred complaint of gait impairment: which procedure is more likely to restore normal function? Indeed, functional studies have shown statistically significant advantages of UKA by having better patient-reported outcome measures and activities of daily living (ADLs) when compared to TKA [11, 12, 27, 28, 30]. But selection bias has always been a concern, as younger, lower body mass index (BMI) and motivated patients tend to be offered UKA [14, 15]. To offset that concern of perceived advantage and to ensure patient parity, it was surmised that patients with a well-functioning partial knee on one side and a well-functioning total knee arthoplasty on the contralateral side of the knee would help give an indication if in fact it matters at all. The primary aim of this study was, therefore, to compare how patients with both a UKA and TKA on either side load their limbs at an everyday walking pace and incline. The secondary aim was to compare these loading patterns with two control groups: healthy controls with no known joint disease, and patients with unilateral medial unicompartmental OA awaiting surgery. The null hypothesis was that no difference would be detected between the way patients load the limb with well performing UKA and the TKA on either side.

## Method

### Participants

**Fig. 1:**
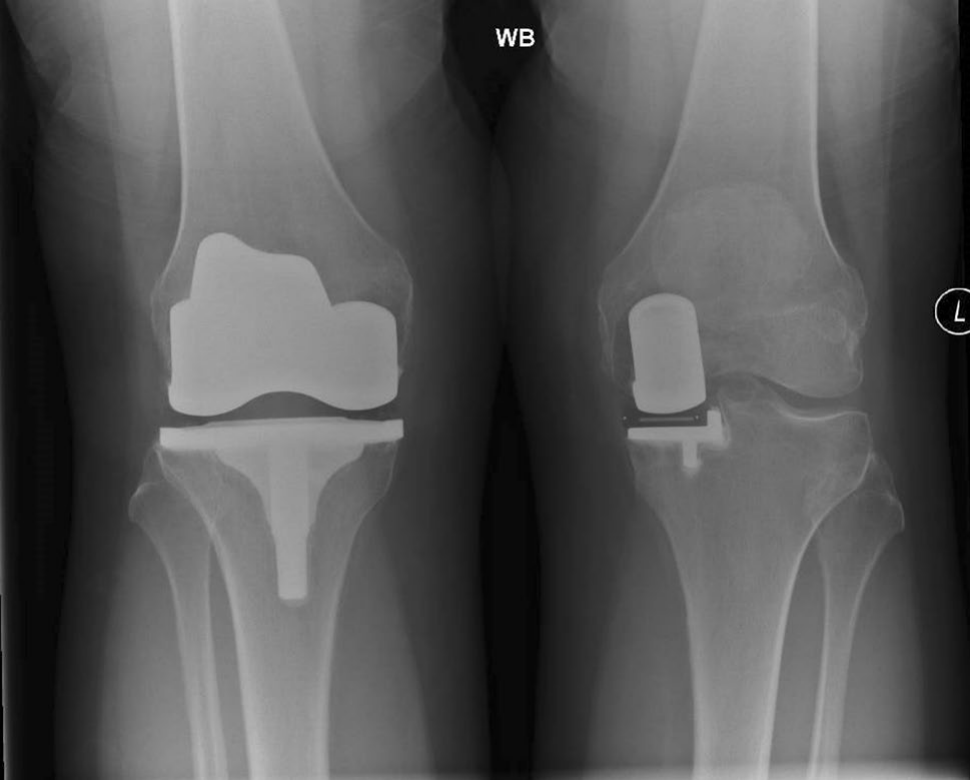
The image shows a postoperative weight bearing plain radiograph after differing knee arthroplasty

Ethical approval in accordance with the ethical standards in the 1964 Declaration of Helsinki was obtained prior to commencement of this study reporting function. The audit retrospectively identified all knee arthroplasty patients who had one UKA and one TKA on contralateral sides. Patients at least 6 months following the most recent surgery were identified from the surgical databases of three senior knee surgeons with a combined 70 year experience in both procedures which they do regularly. All three surgeons use a medial parapatellar approach and instrument the implants as guided by the company operational technique. The Oxford knee score questionnaire was collected for both knees rather than individually to prevent inducing conscious bias toward a particular limb. Pre-operative and post-operative radiographic analysis was undertaken to determine extent of OA disease and ensure satisfactory implant alignment, respectively (Fig. 1). Patient notes were examined to ensure no further surgery and range of motion at discharge.

Between 2000 and 2015 a total of 57 patients were identified by the clinical governance department who had undergone one of each procedure. After imaging review, 22 patients were automatically excluded due to other joint disease or arthroplasty procedures. This left 35 patients to be contacted by the research coordinator who removed 15 due to a safety exclusion protocol of our study ethics having had previous stroke (*n* = 2), unstable heart disease (*n* = 3), lung disease (*n* = 2), spine disease (*n* = 2), metastatic cancer (*n* = 3), and being uncontactable (*n* = 3). Twenty subjects agreed to take part, for which 2 never came for unknown reasons and 2 were unable to walk unaided during gait analysis due to balance difficulty. This left a total of 16 patients who consented to have their gait collected by a blinded assessor.

The arthroplasty subjects had a range of implant types and designs. A total of four different implants were used for the TKA and 2 for the UKA. There were 11 Smith Nephew (Memphis, Tennessee, USA) Genesis II cruciate retaining (all patella resurfaced), 3 MatOrtho (UK) Saiph medial pivot (all patella resurfaced), 1 DePuy (Warsaw, Indiana, USA) PFC Sigma posterior stabilised (patella resurfaced) and 1 Zimmer (Warsaw, Ind, USA) Nexgen cruciate retaining (patella resurfaced) TKA implants. There were 9 Smith Nephew (Memphis, Tennessee, USA) Accuris (two all poly tibia, remaining conventional metal-backed tibial component) and 7 Zimmer Biomet (Warsaw, Indiana, USA) Oxford (mobile bearing) UKA, all on the medial side.

Two further demographically similar groups of subjects with previous gait analysis were obtained from an established treadmill database for comparison. The healthy group of subjects consisted of members of the institution who had no history of joint disease or significant past medical history. The knee OA group consisted of ipsilateral knee OA patients with isolated medial compartmental disease awaiting surgery.

### Gait instrumentation

A validated instrumented treadmill (Gaitway Kistler, Kistler Instrument Corporation, Amherst, NY) with a previously reported protocol, was used to collect patient gait data. It has been shown to be reliable and reproducible [10, 26]. The vertical component of the ground reaction forces (GRF) were collected on calibrated tandem Kistler force plates at a sample frequency of 100 Hz. All participants were weighed with the force plate prior to assessment, to allow normalisation for body weight as according Hof et al. [7] A standardised warmup and acclimatisation period of 6 min were completed before unaided gait collection. Gait collection only occurred after the patient felt a steady state had been achieved. The data collection period lasted 20 s for each condition with level walking tested first, followed by the uphill assessment.

### Gait variables, processing and analysis

To avoid any perceived functional advantage of arthroplasty type, an analysis at a comfortable speed of 4.5 km/h was chosen based on a previously published work [26]. It found knee OA patients preferred walking speed being 4 km/h and controls being 5 km/h. A 5% incline at 4 km/h was also collected as it a common activity of daily living and has been shown to influence the condition to test gait [10]. Ground reaction force (GRF) was the focus of analysis as it reflects the load transmitted through the limb and thus will reveal any preference or limb dominance. Maximum force (Max Force), weight acceptance (WA), midstance (MS) and pushoff (PO) and the difference between weight acceptance and mid stance (WA–MS) were chosen based on previous studies showing an intra-class correlation coefficient of 0.93–0.99, signifying excellent reliability and repeatability [10].

A previously described and validated symmetry ratio (SR) [18] was also applied to the GRF to indicate the direction and percentage difference between limbs. Zero being complete symmetry with positive and negative percentage signifying more or less load to the numerator limb respectively. The limbs were divided into the arthroplasty group as UKA/TKA, the OA group as unaffected/OA affected, the control as right / left limb respectively.

$${\text{SR}} = ((X \cdot {\text{UKA}}/X \cdot {\text{TKA}}) - 1) \times 100\%$$

All trials were visually processed to ensure six consecutive strides were taken cleanly. Typically ten or more strides were collected so a Matlab (Mathworks, Mass, USA) script was written to extract the data from the Kistler software in a formatted manner for analysis. Statistical analysis was done with Matlab. Shapiro–Wilk test showed a normal distribution, therefore parametric tests were used. To determine difference between groups, a one-way analysis of variance (ANOVA) with Tukey post-hoc test was used, with significance set at *p* < 0.05 throughout. Paired *T*-tests were carried out to detect significant differences in GRF in the UKA and TKA limbs in the arthroplasty group and the knee OA limb compared to the unaffected limb in the knee OA group.

A minimum sample size of 9 was chosen based on a previous gait study with an analogous design comparing hip resurfacing and total hip arthroplasty, which showed a statistical and minimum clinical difference of 5% [1].

## Results

A total of 48 subjects, comprising 16 in each group, had analysis. All three groups were similar for gender, age, height and BMI (Table 1). The arthroplasty group had 9 TKA and 7 UKA on the right. In 7 patients, the TKA was the first procedure, while 6 for the UKA and 3 done simultaneously. The mean time from surgery to gait assessment was 38 months for the UKA and 45 months for the TKA. All knee arthroplasty patients were content and without reoperation at the time of gait assessment with a mean Oxford Knee Score (OKS) of 41 (37–48). Preoperatively, the most common Kellgren–Lawrence grade for each knee radiographically was 3 with all knees having isolated medial compartment disease except 3. One pair of knees was wind swept (varus and valgus alignment) and the two others had patellofemoral wear on one side. Postoperatively, there were no clinical implant malalignment (Table 1) or evidence of loosening at final radiographic review. The mean range of motion for the TKA side was 108° (95–120) and 115° (100–120) for the UKA side.

**Table 1:** Demographic data

Subject	Control	UKA	TKA	OA
Sex M:F	7:9	7:9		7:9
Age (years)	67 (10)	70 (8)		66 (7)
BMI	26 (4)	30 (3)		29 (6)
Height (cm)	167 (9)	168 (10)		171 (7)
Pre-Op OA severity (Kellgren–Lawrence)	NA	3 (2–4)	3 (2–4)	3 (2–4)
Implanted side (right)	NA	7	9	NA
Gait follow-up (months)	NA	38 (8–108)	45 (8–180)	Pre-op
Coronal femoral angle	NA	7 (3–10)	7 (4–10)	NA
Coronal tibial angle	NA	86 (84–90)	89 (84–92)	NA
Sagittal tibial posterior slope	NA	4 (2–7)	5 (1–8)	NA

Mean results (standard deviation—except for range for gait followup period and implant alignment degrees)

### Gait

**Fig. 2:**
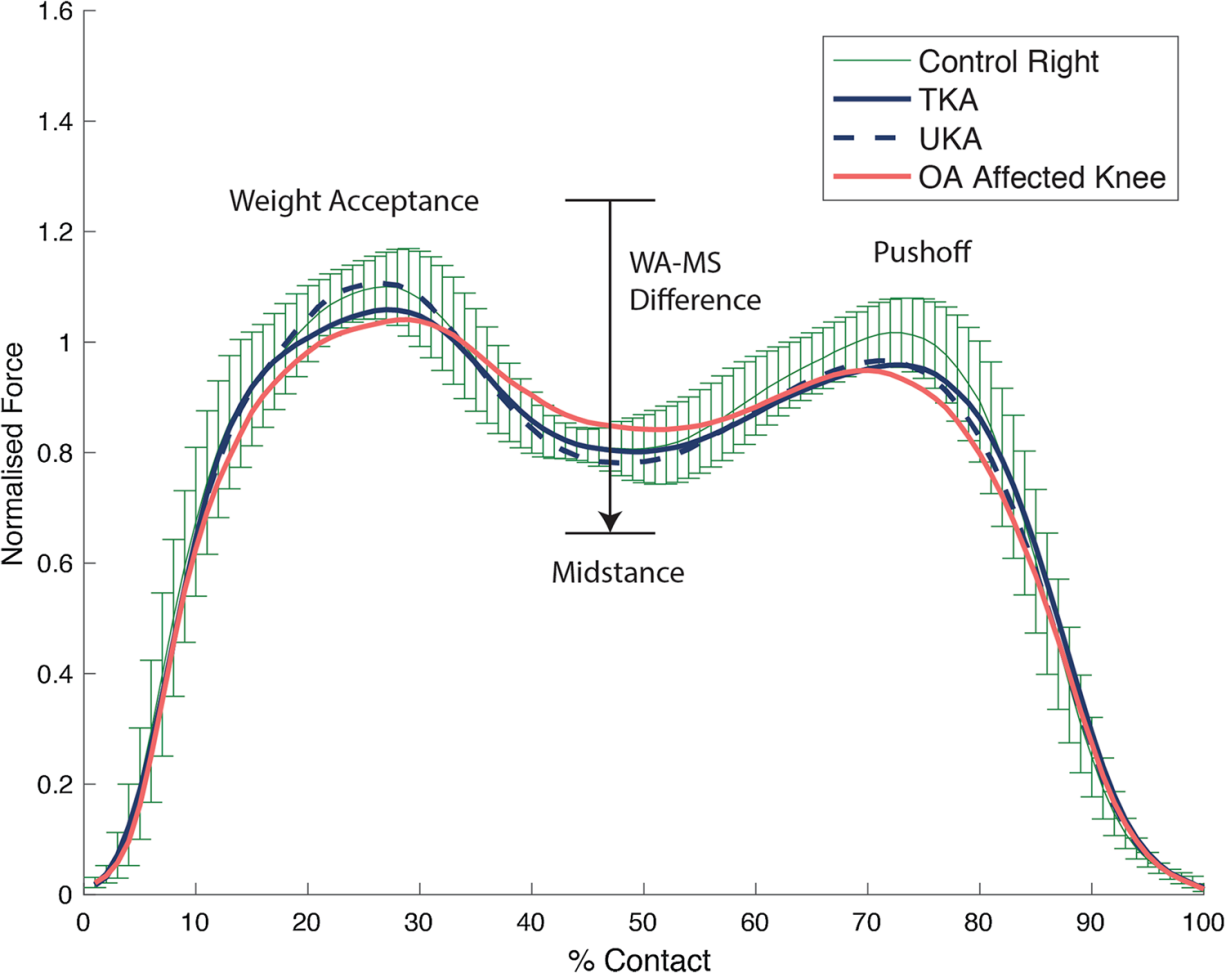
The graph shows the mean normalised ground reaction forces along with the 95% confidence interval for the controls during level walking at 4.5 km/h. *TKA* total knee arthroplasty, *UKA* unicompartmental knee arthroplasty, *OA* osteoarthritis, *WA–MS* weight acceptance–midstance difference

**Fig. 3:**
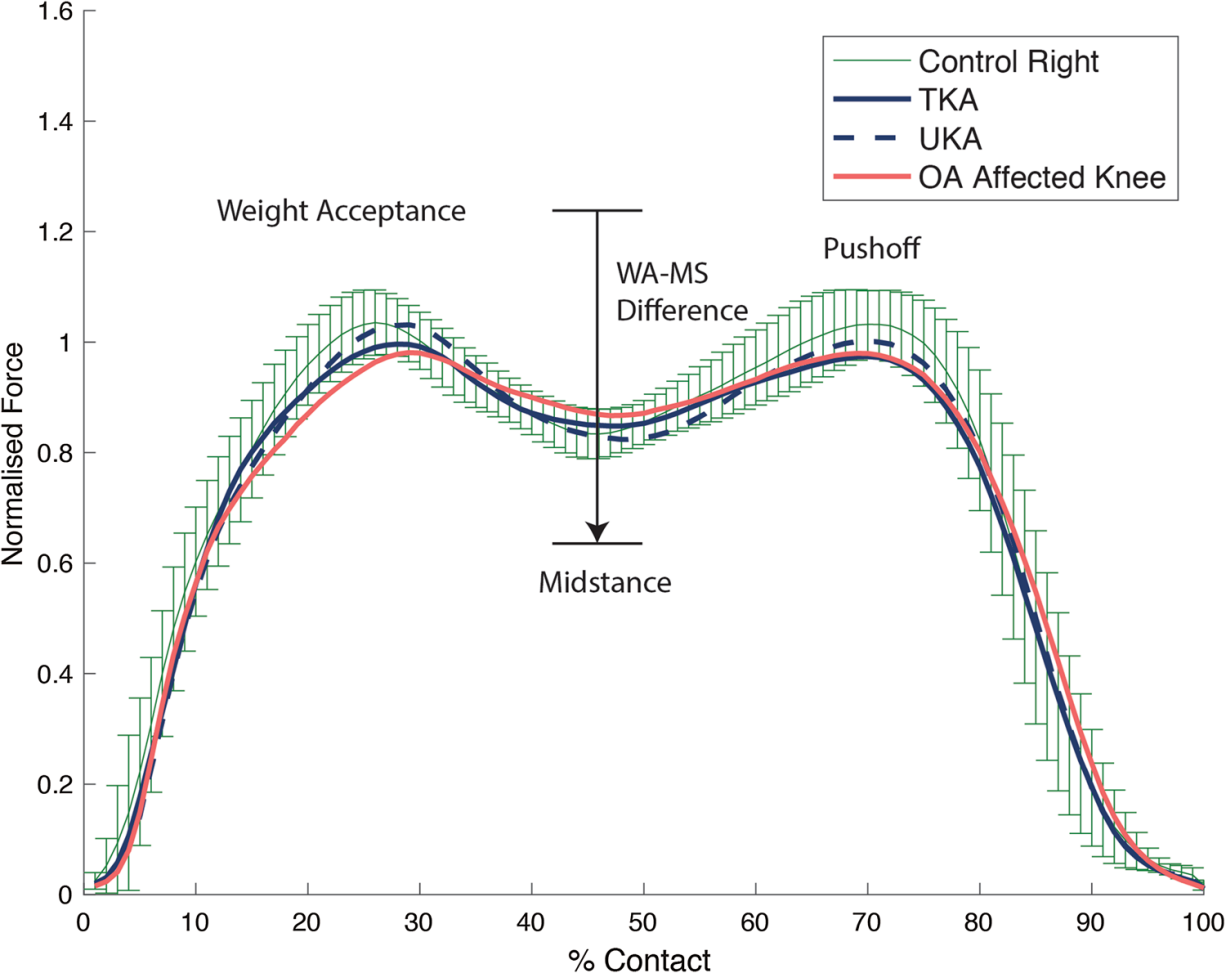
The graph shows the mean normalised ground reaction forces along with the 95% confidence interval for the controls during incline walking at 4 km/h. *TKA* total knee arthroplasty, *UKA* unicompartmental knee arthroplasty, *OA* osteoarthritis, *WA–MS* weight acceptance–midstance difference

Healthy controls predictably walked with a symmetrical gait (Tables 2 and 3). During level walking the right limb was the preferred side and during uphill walking it was the left. During both level and uphill walking (Tables 2 and 3), knee OA subjects walked with a significantly asymmetrical pattern (*p* = 0.01–0.00001). Significantly higher force was transmitted through the unaffected limb, sparing the arthritic knee during concentric loads whereas during midstance the knee OA limb failed to offload suffering significantly higher forces. In comparison to healthy controls, despite clearly putting abnormal forces through the limb the knee OA group was not statistically dissimilar and only reached significant difference on the OA limb side during midstance and WA–MS difference during level walking (*p* = 0.007 and *p* = 0.010 respectively). In the knee arthroplasty group, higher forces except midstance were transmitted through the UKA limb in all cases (Figs. 2 and 3), on both during flat and incline walking. During flat walking, the UKA limb was closer to normal during max force (*p* = 0.022) weight acceptance (*p* = 0.049), midstance (*p* = 0.004), WA–MS difference (*p* = 0.008) and uphill during max force (*p* = 0.013) and WA–MS difference (*p* = 0.045). The greatest variance was detected by the WA–MS difference, a 22% difference was noted between limbs in both gait conditions in favour of the UKA side. When analysing each individual’s variables in isolation during level and incline walking, the UKA limb was the preferred side 76% of the time, whilst 24% for the TKA limb. In comparison to controls, the only elements of TKA limbs function which were significantly inferior to the controls while flat walking were MS (*p* = 0.021) and WA–MS difference (*p* = 0.019) while any differences seen in other variables failed to reach significance. The limb with the UKA was not distinguishable from the healthy controls using any variable.

**Table 2:**
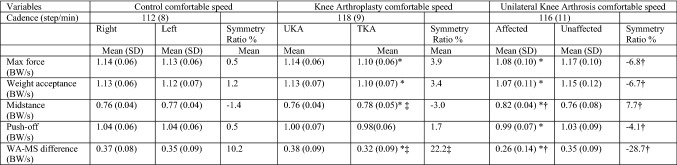
Normalised ground reaction results during level walking

*BW *Body weight normalised

The values are indicated as means (standard deviation); ^†^significant difference between OA group versus control (*p* < 0.05)**; **^‡^ significant difference between knee arthroplasty versus control; *significant difference between limbs in individual groups

**Table 3:**
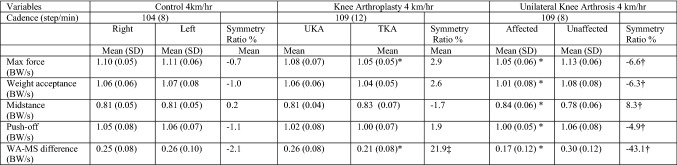
Normalised ground reaction results during 5% incline walking

*BW* Body weight normalised

The values are indicated as means (standard deviation); ^†^significant difference between OA group versus control (*p* < 0.05); ^‡^significant difference between knee arthroplasty versus control; *significant difference between limbs in individual groups

## Discussion

The most important finding of this gait study was that the null hypothesis was over-turned. The loading pattern of the UKA limb resembled the healthy controls significantly more closely than the TKA limb. The UKA limb was able to load and offload 22% more physiologically than TKA during the transition between weight acceptance and midstance. The TKA limb, on the contrary, were significantly inferior during this transition (*p* = 0.019) and in midstance (*p* = 0.021) closely resembling the forces seen in patients awaiting arthroplasty with knee OA. Interestingly this observation has been reported in patients with absent anterior cruciate ligaments (ACLs) with instability symptoms and patients with knee OA [9, 22], where co-contraction of the quadricep-hamstring pair during midstance in times of instability or pain is termed ‘stiffening strategy’. This implies that the TKA limb was more unstable than the UKA limb, as pain was not reported during the gait assessment. Paradoxical motion and midflexion instability as found after ACL removal is a documented limitation of TKA [3, 24, 25]. The UKA limb, on the contrary had no difficulty transitioning through the phases of loading and was indistinguishable from healthy controls in all variables except push-off, which is consistent with a previous study comparing TKA and UKA cohorts during downhill walking [27]. Another interesting finding was that of laterality: the UKA limb was the favoured limb 76% of the time irrespective of variable and gait condition. It is well documented that small asymmetries exist in the normal population due to limb dominance as one is being used for propulsion related tasks and the other for locomotor balance [20, 21]. The right in the literature has been the power generator and the left for targeting [19]. In our arthroplasty cohort, 9 TKA was implanted on the right whereas 7 for UKA. Considering this, the fact that 12 of the 16 patients preferred the UKA limb suggests that an overwhelming performance shift of the limbs in preference to the UKA side.

The principle limitation of this gait study is the lack of randomisation which could result in a selection bias. In such a small and random sample, an unknown bias may also exist. The small sample size is not surprising—only a very few patients have unorthodoxically received two different types of arthroplasty in differing knees for essentially the same condition. While lacking randomisation, fortuitously, there was a similar ratio of arthroplasty types in terms of first procedure and laterality. Additionally, all implants in the study group were highly rated devices as by ODEP (Orthopaedic Data Evaluation Panel) with no less than an “A” rating. The range of implants helps ensure some pragmatism, focusing on a surgical philosophy and not of a particular device advantage. Another limitation is only testing two comfortable condition ADLs rather than a range of more taxing activities. This was purposeful as the knee OA patients were essential for the analysis. It was important to understand the bottom range of normality and assessing higher function would not be possible with them. This method observed the possibilities of the implanted knees without any perceived advantage and represents what patients could expect after having an operation type even if they were not ambitious.

The strengths of the study included the analysis of matched subjects at each end of knee health with the normal controls and knee OA patients as control groups. This select group of patients with an assessment more than 3 years after arthroplasty likely represents a reasonable alternative to a randomised comparative study as it let the patient decide, without external influence, how the knee is loaded. The two gait conditions confirmed the primary hypothesis that there is a measurable difference between the load transfer following these two arthroplasty philosophies. The secondary aims were also met: when compared with healthy controls and patients awaiting knee surgery for OA, the UKA limbs transmitted forces that closely resembled healthy controls at heel strike and in midstance. Yet at push-off the UKA could not match the controls which continue the quest to improve function. The TKA limbs did not fare as well, and in some areas of function, such as midstance, they more closely resembled patients with OA of the knee awaiting surgery. These findings may aid the decision making tree to better inform surgeons and patients alike.

## Conclusion

This small study suggests that UKA does indeed enable a more natural gait than TKA when ground reaction forces on a treadmill with differing inclines are used as surrogates for function. The decision of which procedure to choose should not be made on these grounds alone, but should also include considerations regarding patient safety.
